# Immunologic Changes Implicated in the Pathogenesis of Focal Segmental Glomerulosclerosis

**DOI:** 10.1155/2016/2150451

**Published:** 2016-02-17

**Authors:** Andreas Kronbichler, Johannes Leierer, Jun Oh, Björn Meijers, Jae Il Shin

**Affiliations:** ^1^Department of Internal Medicine IV (Nephrology and Hypertension), Medical University Innsbruck, Anichstraße 35, 6020 Innsbruck, Austria; ^2^Pediatric Nephrology, University Medical Center Hamburg-Eppendorf, Martinistraße 52, 20246 Hamburg, Germany; ^3^Department of Nephrology, UZ Leuven, Leuven, Belgium; ^4^Department of Immunology and Microbiology, KU Leuven, Leuven, Belgium; ^5^Department of Pediatric Nephrology, Severance Children's Hospital, Yonsei University College of Medicine, Seoul, Republic of Korea

## Abstract

Focal segmental glomerulosclerosis is a histological pattern on renal biopsy caused by diverse mechanisms. In its primary form, a circulatory factor is implicated in disease onset and recurrence. The natural history of primary FSGS is unpredictable, since some patients are unresponsive towards immunosuppressive measures. Immunologic changes, leading to a proinflammatory or profibrotic milieu, have been implicated in disease progression, namely, glomerular scarring, eventually leading to end-stage renal disease. Among these, interleukin-1ß, tumor-necrosis factor-*α* (TNF-*α*), and transforming growth factor-ß1 (TGF-ß1) have emerged as important factors. Translating these findings into clinical practice dampened the enthusiasm, since both TNF-*α* and TGF-ß1 blockade failed to achieve significant control of the disease. More recently, a role of the complement system has been demonstrated which in fact may be another attractive target in clinical practice. Rituximab, blocking CD20-bearing cells, demonstrated conflicting data regarding efficacy in FSGS. Finally, the T-cell costimulating molecule B7-1 (CD80) is implicated in development of proteinuria in general. Blockade of this target demonstrated significant benefits in a small cohort of resistant patients. Taken together, this review focuses on immunology of FSGS, attributable to either the disease or progression, and discusses novel therapeutic approaches aiming at targeting immunologic factors.

## 1. Introduction

Primary or idiopathic focal segmental glomerulosclerosis (FSGS) remains a therapeutic challenge due to its unpredictable disease course. It is assumed to be a lesion rather than a specific glomerular disease and different lesions have been described by the Columbia group which may predict renal outcome of the patients [1]. In primary FSGS one or more putative circulatory factor(s), yet to be identified, are implicated in disease occurrence and recurrence. Immunologic changes attributable to primary FSGS have attracted more attention recently, since targeted therapeutics became available over the last two decades. The aim of this review is to focus on immunologic changes described in primary FSGS and their implication on potential future therapeutic options.

## 2. T-Cell Involvement in Focal Segmental Glomerulosclerosis

Early reports hypothesized whether T-cell dysfunction is implicated to play a role in FSGS or not. In a small cohort of patients with FSGS, a normal distribution of CD3+, CD4+, and CD8+ T-cells was found [2]. Another investigation highlighted abundant expression of CD8+ T-cells, whereas CD4+ T-cell count was reduced compared to age-matched controls [3]. The latter was accompanied by an increase in interleukin-2R (IL-2R, CD25) expression on CD4+ cells [3]. CD3-staining of kidney biopsies revealed significantly higher levels in FSGS compared to minimal change disease (MCD) or controls. In contrast, FoxP3+ regulatory T-cells were decreased in FSGS and MCD compared to control biopsies [4] and may increase once remission is achieved [5]. Restoration of FoxP3+ regulatory T-cells was associated with regression of nephropathy in a rat model [6].

Circulating Th17 cells as assessed in peripheral blood mononuclear cells (PBMC) were more abundant in patients with nephrotic syndrome compared to controls and were higher in non-MCD patients. A role for the Th17/interleukin-17 (IL-17) axis was further supported by the finding that IL-17 staining was most abundant in FSGS biopsies compared to MCD and mesangial proliferative glomerulonephritis. In addition,* in vitro* studies revealed a time- and dose-related proapoptotic effect of IL-17 on podocytes [7]. Interleukin-4 positive T-helper cells (Th2) did not differ between FSGS and MCD patients, whereas a significantly higher amount was present in patients with membranous nephropathy. In contrast, the peripheral Th1/Th2 ratio (IFN-*γ*/IL-4 ratio) was significantly lower in membranous nephropathy when compared to the other entities. Proteinuria correlated with the expression of IL-4 positive cells [8].

## 3. B7-1 (CD80) and Focal Segmental Glomerulosclerosis

The observation that podocyte expression of the T-cell costimulatory molecule B7-1 (CD80) may be induced during glomerular injury, while being absent in normal kidneys, promoted further explorations in patients with FSGS. In a small cohort of patients with either naïve or recurrent FSGS, positive B7-1 staining was present. In an analysis of diverse glomerular pathologies, patients with lupus nephritis showed the strongest glomerular or mesangial B7-1 staining [9]. However, a subsequent study did not confirm any positive podocyte expression of CD80 in patients with FSGS. Benigni and colleagues failed to show staining of B7-1 in naïve or recurrent FSGS [10]. In order to understand the role of B7-1 in the development of FSGS further studies are required.

## 4. Complement

The complement system is involved in several glomerular diseases. Recently, Thurman and colleagues analyzed samples from patients with FSGS enrolled in a study comparing efficacy of cyclosporine A (CSA) with mycophenolate mofetil (MMF). Patients with FSGS had higher levels of plasma Ba and C4a compared to healthy controls and patients with antineutrophil cytoplasm antibody- (ANCA-) associated vasculitis or lupus nephritis or healthy individuals. Urinary C4a levels were highest in FSGS patients compared to samples obtained from patients with chronic kidney disease (CKD), ANCA-associated vasculitis, and lupus nephritis. Both plasma and urine sC5b-C9 were significantly higher in patients with FSGS compared to the comparators including CKD patients. Although number of subgroup analyses is low, MMF-treated patients showed a significant decline of plasma sC5b-C9 over time [11].

## 5. Transforming Growth Factor-ß1

Intrarenal gene expression of transforming growth factor-ß1 (TGF-ß1) revealed a positive predictive value of 90% and a negative predictive value of 80% to identify FSGS compared to other examined histologic lesions. Among the cytotoxic effectors, Fas ligand tended to show coexpression with TGF-ß1, while granzyme B and perforin were expressed in all steroid-resistant cases [12]. In line with this observation, Souto and coworkers showed abundant expression of TGF-ß1 in steroid-resistant cases (majority having FSGS) compared to controls. Moreover, TGF-ß1 expression was highest in patients with a relapsing steroid-resistant disease course [13].* In vitro* experiments indicated an upregulation of neuropilin-2 (NRP2) following TGF-ß1 stimulation, which was inversely correlated with estimated glomerular filtration rate at the time of biopsy and correlated with subsequent decline in renal function [14]. This highlights a role of TGF-ß1 in FSGS, especially in those with a steroid-resistant disease course who might progress to end-stage renal disease (ESRD).

## 6. Other Cytokines and Their Role in Focal Segmental Glomerulosclerosis

Other cytokines, namely, interleukin-1ß (IL-1ß) and interleukin-6 (IL-6), were elevated in patients with nephrotic syndrome compared to controls. Renal histopathology revealed higher IL-1ß expression in FSGS kidney biopsy specimen compared to MCD or mesangial proliferative glomerulonephritis [7]. Immunohistochemistry highlighted a differential regulation of glomerular and tubulointerstitial expression of tumor-necrosis factor-*α* (TNF-*α*) in MCD and FSGS. Glomerular staining for TNF-*α* expression was scarce, while tubulointerstitial staining was prominent in FSGS. This was contrary in patients with MCD. Bakr et al. reported on children with MCD and FSGS. TNF-*α* levels were significantly higher in patients with active nephrotic syndrome and correlated with the degree of proteinuria. Moreover, positive correlation between TNF-*α* production and the degree of mesangial hypercellularity and glomerulosclerosis was reported. A TNF-*α* level of greater than or equal to a cut-off of 50 pg/mL was able to predict resistance towards steroids in these patients (predictability 93.2%) [15].

Interleukin-10 (IL-10) levels were almost identical in both entities and increased during nephrotic-range proteinuria. The authors speculated that IL-10 is increasing with the amount of protein loss, whereas TNF-*α* in the tubulointerstitium may reflect interstitial fibrosis [16]. Serum interleukin-12 (IL-12) was not detectable in the majority of patients with FSGS [17]. Niemir and coworkers observed differential expression in preserved glomeruli compared to sclerotic ones. Whereas IL-1*α*/ß, IL-1 RII, and IL-1 receptor antagonist (RA) were similarly distributed in nonsclerotic glomeruli of patients with FSGS, glomerulosclerosis was accompanied by a scarce expression of IL-1ß and IL-1 RII only [18]. Analysis of urinary cytokine excretion revealed significantly higher levels of interleukin-2 (IL-2), interleukin-4 (IL-4), IL-6, IL-10, interferon-*γ* (IFN-*γ*), and monocyte chemoattractant protein-1 (MCP-1) in a subgroup of patients with MCD/FSGS, whereas interleukin-17A (IL-17A), TNF-*α*, and TGF-ß1 were unaltered compared to a control group [19]. Urinary excretion of interleukin-18 (IL-18)/CXCL8, MCP-1/CCL2, and RANTES/CCL5 was not different in an analysis of patients with steroid-resistant or steroid-sensitive nephrotic syndrome. However, there was an association between IL-18/CXCL8 expression and degree of proteinuria [13].

## 7. Macrophages, HLA, and Myeloid-Derived Suppressor Cells in Focal Segmental Glomerulosclerosis

Interstitial staining for CD68 implicated a significantly higher number of macrophages in childhood FSGS compared to MCD or control biopsy specimens. Patients with a steroid-resistant course had higher numbers compared to steroid-dependent or frequently relapsing patients [4]. Examination of human leukocyte antigen (HLA) by immunohistochemistry indicated that the HLA-DR antigen was present in all patients in glomerular endothelial cells, whereas positivity was present in one quarter in extraglomerular mesangium cells and podocytes [20]. Reduction of diverse HLA class II antigens, namely, −DQ, −DR, −DP, and –DY, was observed in sclerotic glomeruli of patients with FSGS in comparison to healthy kidney tissue [21]. Myeloid-derived suppressor cells (MDSC), characterised by CD11b+HLA-DR-CD14-CD15+ staining, in peripheral blood increased following initiation of steroids in responsive FSGS subjects, whereas no increase was found in steroid-resistant patients. Induction of MDSC was capable of suppressing T-cell proliferation and induced regulatory T-cells* in vitro* [22].

## 8. B-Cells and Focal Segmental Glomerulosclerosis

Glomerular staining for CD20 positivity was significantly and numerically higher in FSGS compared to controls and MCD, respectively. In contrast, interstitial staining was reduced in FSGS and MCD in comparison to control biopsies [4]. Strassheim and coworkers examined the effect of anti-CD20 treatment and prevention of IgM deposition in a mouse model of FSGS. Approximately 30% of the kidney biopsies examined displayed glomerular IgM deposition, either colocalized with C3 or not. This subgroup may be more susceptible towards a B-cell depleting therapy as shown in their Adriamycin-induced nephropathy [23].

## 9. Transition into Clinical Practice: Towards Tailored Medicine in Focal Segmental Glomerulosclerosis

### 9.1. Abatacept

Yu and coworkers demonstrated efficacy of B7-1 blockade with abatacept (CTLA-Ig) in rituximab- and steroid-resistant cases [9]. Mechanistically, they found that abatacept was capable of blocking ß1-integrin activation. Although these data suggest clinical benefit based on mechanistic insights, the initial enthusiasm was seriously dampened by subsequent reports. Proteinuria remained unchanged in one patient with primary and three patients with recurrent FSGS receiving either abatacept or belatacept, the latter having predominant effects on B7-2 (CD86) [24]. Although kidney biopsies from patients with lupus nephritis showed strong B7-1 staining [9, 25], a recent trial comparing abatacept as add-on therapy to cyclophosphamide to a standard-of-care treated control group showed no improvement with the addition of abatacept, again disproving a role of B7-1 blockade in proteinuric kidney diseases [26].

### 9.2. Adalimumab

As demonstrated above, TNF-*α* is a promising target in patients with FSGS. Thus, adalimumab, a human monoclonal antibody targeting TNF-*α*, was tested in a phase I trial including 10 patients with resistant FSGS. Pharmacokinetics revealed an increased clearance by 160% compared to healthy controls and patients with rheumatoid arthritis [27] which was attributable to renal and nonrenal clearance with a direct impact of proteinuria on the former finding [28]. While two patients had a partial remission during follow-up time (proteinuria decreased from a PCR of initially 3.6 and 17 mg/mg creatinine to 0.6 in both subjects), the other eight patients remained nephrotic [27]. Moreover, the authors demonstrated a stabilization of the estimated glomerular filtration rate during follow-up in some patients [29]. A subsequent phase II trial in resistant FSGS failed to recruit significant numbers. Of the seven included patients, no patient had a significant response with worsening of proteinuria in 4/6 patients [30]. Although small numbers limit definite conclusions, a general recommendation related to adalimumab use in resistant FSGS is not warranted.

### 9.3. Fresolimumab

TGF-ß is implicated in several mechanisms leading to pathologic glomerular changes. Targeting this pivotal cytokine with a human monoclonal antibody, namely, fresolimumab, led to an estimated glomerular filtration rate decline of 5.9 mL/min (annualized 19 mL/min), whereas mean proteinuria measured as PCR decreased by 1.2 mg/mg creatinine in a phase I trial [31]. A further multinational trial investigating the efficacy of this substance has already completed recruitment.

### 9.4. Rituximab

Although experience of rituximab's use in FSGS is limited to case reports or series, it is widely used to treat resistant cases [32]. Analysis of the Spanish Group for the Study of Glomerular Diseases (GLOSEN) revealed that two patients had a clear and sustained improvement following rituximab treatment, while one patient had a transient response twice and the other five patients did not respond to the treatment [33]. In our analysis including relapsing patients most patients achieved sustained remission with a reduction of relapses following rituximab. However, one patient showed no response following rituximab therapy [34]. This is in line with a recent analysis of childhood onset steroid-resistant and congenital nephrotic syndrome, which showed equivalent response rates of rituximab compared to calcineurin inhibitors, with 40–45% of the patients achieving complete remission [35]. Treatment with rituximab might lead to life-threatening infections as reported in ANCA-associated vasculitis, for example [36]. No such complications have been reported in adult patients with FSGS treated with rituximab so far. Thus, it might be an option in difficult-to-treat FSGS [37]. One potential para-CD20 effect of rituximab may in fact be stabilization of the actin cytoskeleton. Rituximab partially prevented sphingomyelin phosphodiesterase acid-like 3b (SMPDL-3b) downregulation and prevented disruption of the actin cytoskeleton along with apoptosis of podocytes induced by FSGS sera [38].

## 10. Conclusions

Several immunologic changes have been identified during the last decades in FSGS. However, lack of replication or failure of translation into useful therapeutic measures limits these findings. With improved laboratory techniques novel potential targets will be elucidated in the future and hopefully therapeutic concepts targeting specific molecules, such as rituximab's effects on SMPDL-3b, will emerge. Overall, the aim has to be identification of the responsible pathogenic factor(s), which may in fact be a useful marker as has been shown in idiopathic membranous nephropathy.
